# Dynamic Control of Selectivity in the Ubiquitination Pathway Revealed by an ASP to GLU Substitution in an Intra-Molecular Salt-Bridge Network

**DOI:** 10.1371/journal.pcbi.1002754

**Published:** 2012-11-01

**Authors:** Sjoerd J. L. van Wijk, Adrien S. J. Melquiond, Sjoerd J. de Vries, H. Th. Marc Timmers, Alexandre M. J. J. Bonvin

**Affiliations:** 1 Department of Molecular Cancer Research, Division of Biomedical Genetics and Netherlands Proteomics Center, University Medical Center Utrecht, Utrecht, The Netherlands; 2 Computational Structural Biology Group, Bijvoet Center for Biomolecular Research, Faculty of Science, Utrecht University, Utrecht, The Netherlands; Stanford University, United States of America

## Abstract

Ubiquitination relies on a subtle balance between selectivity and promiscuity achieved through specific interactions between ubiquitin-conjugating enzymes (E2s) and ubiquitin ligases (E3s). Here, we report how a single aspartic to glutamic acid substitution acts as a dynamic switch to tip the selectivity balance of human E2s for interaction toward E3 RING-finger domains. By combining molecular dynamic simulations, experimental yeast-two-hybrid screen of E2-E3 (RING) interactions and mutagenesis, we reveal how the dynamics of an internal salt-bridge network at the rim of the E2-E3 interaction surface controls the balance between an “open”, binding competent, and a “closed”, binding incompetent state. The molecular dynamic simulations shed light on the fine mechanism of this molecular switch and allowed us to identify its components, namely an aspartate/glutamate pair, a lysine acting as the central switch and a remote aspartate. Perturbations of single residues in this network, both inside and outside the interaction surface, are sufficient to switch the global E2 interaction selectivity as demonstrated experimentally. Taken together, our results indicate a new mechanism to control E2-E3 interaction selectivity at an atomic level, highlighting how minimal changes in amino acid side-chain affecting the dynamics of intramolecular salt-bridges can be crucial for protein-protein interactions. These findings indicate that the widely accepted sequence-structure-function paradigm should be extended to *sequence-structure-dynamics-function* relationship and open new possibilities for control and fine-tuning of protein interaction selectivity.

## Author Summary

During their life, proteins undergo various modifications ranging from structural marking or signaling to degradation. One major biochemical process involves ubiquitin, a small and evolutionary conserved protein. This regulatory protein serves as a tag that, when attached to a protein substrate, alters its function, cellular sub-location or commits the labeled protein to destruction in the proteasome. The high specificity of the ubiquitination pathway is achieved through interactions between two large protein families, E2 and E3, that ensure the efficient covalent conjugation of ubiquitin. By comparing two “almost identical” E2 enzymes, we identified a single minute substitution that, operated by a dynamic network of salt-bridges, functions as a subtle switch that controls interaction selectivity toward E3 proteins. Using a combination of bioinformatics and modeling techniques, complemented by mutagenesis and experimental screening of E2-E3 interactions, we unraveled an equilibrium between an “open”, binding-competent and a “closed”, binding-incompetent state. Subtle modifications in this network are sufficient to switch the selectivity profile. These findings should serves as a cautionary tale and raises new challenges for bioinformatics analysis, modeling and experimental engineering of protein-protein interactions. The dynamic nature of the identified regulatory switch suggests that the widely accepted sequence-structure-function paradigm should be extended to *sequence-structure-dynamics-function*.

## Introduction

Biological systems critically rely on selective and specific protein interactions, creating building blocks that cooperatively form the basis of functional complexity [1]. In particular, regulatory and signaling pathways, such as conjugation of ubiquitin (Ub) or similar ubiquitin-like modifiers (UBLs), depend on specific recognition of binding partners whilst discriminating against non-specific interactions [2], [3]. Ub becomes conjugated to substrates through the action of the E1-E2-E3 enzymatic system. This cascade follows a pyramidal hierarchy wherein two human Ub-activating enzymes (E1s) and over 30 human Ub-conjugating (E2) enzymes and hundreds of E3 Ub protein ligases cooperate to catalyze subsequent substrate modification [4], [5]. Whereas the Ub-E1 enzymes have different selectivity [6], [7], it is mainly the selective associations between E2 and E3 enzymes among thousands of possible interactions that are primarily responsible for effective Ub conjugation [2], [3], [8], [9].

The molecular basis for this selectivity is provided by surface residues of E2 enzymes and by “cross-braced”, zinc-binding domains of the Really Interesting New Gene (RING) sub-family of E3 ligases [2]. These RING finger domains generally mediate binding with E2s through their highly conserved UBC-fold, although exceptions have been described [8], [10]. This fold is a strictly conserved structure surrounding the active site cysteine that covalently accommodates the activated Ub [8], [11], [12]. Regarding the E2 enzymes, the major structural determinants for E3 binding have been identified in residues located in helix 1 (H1), loop 1 (L1) and loop 2 (L2) within the classical E2-E3 interaction interface [10].

Efficient ubiquitin-conjugation depends on specific interactions between E2 and E3 enzymes. The high degree of sequence and structure conservation in E2s and, to some extent, among the E3 RING-finger domains and their reported E2-E3 interfaces, remains compatible with a highly selective binding for their cognate E2 or E3 partners [9], [13]. Conserved sub-families of E2 enzymes, such as those belonging to the UbcH5 branch, have been shown to cooperate with similar sub-sets of E3 ligases [13]. At the other side, structurally more divergent E2s display interactions with more specialized E3 enzymes, such as for example the APC/C-UbcH10 pair [14]. Furthermore, several studies have indicated that the ability of E2-E3 pairs to mediate biochemical Ub- or UBL-conjugation is directly dependent on the ability of physical E2-E3 interactions [15], [16].

Based on this, physical E2-E3 interactions can be regarded as primary determinants for efficient conjugation reactions. We demonstrate here that a subtle and minute change – an aspartic to glutamic acid substitution (one methylene group difference) – is sufficient to completely change the selectivity profile of an E2 enzyme toward E3 RING-finger domains. Using molecular modeling and molecular dynamics simulations, a network of intra-molecular salt-bridges was identified that controls the balance between a binding-competent and a binding-incompetent state. Perturbation of network components located both in and outside the classical interaction surface resulted in a switch in the E3 interaction binding profile. These results suggest a new and delicate mechanism of how protein-protein interaction selectivity is achieved within the promiscuous ubiquitination system.

## Results

**Figure 1:**
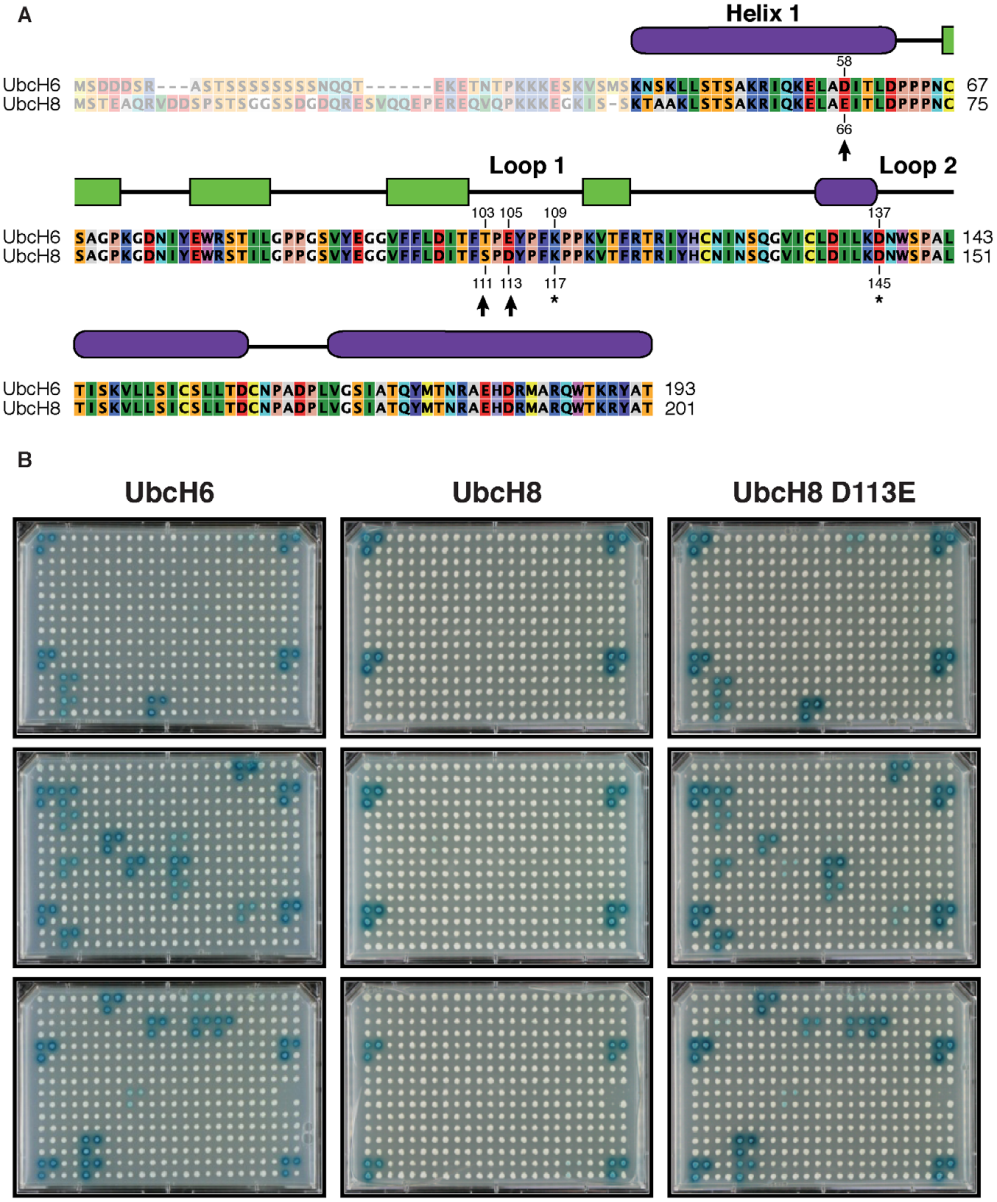
The highly similar UbcH6 and UbcH8 E2 enzymes interact with different RING E3 domains cohorts. **A.** Sequence alignment of human UbcH6 (UBE2E1) and UbcH8 (UBE2E2). Secondary structures and E3-interface regions (H1, L1 and L2) are indicated. Arrows indicate E2-specific residues; asterisks indicate conserved residues involved in bridging. N-terminal extensions are indicated as shaded. **B.** E3-interaction profiles of *wt* UbcH6, UbcH8 and UbcH8 D113E (see Materials and Methods section for details).

In previous studies, global E2-E3 (RING) interactions have been mapped by high-throughput yeast two-hybrid screening (Y2H), revealing that E2 enzymes that show high conservation within their E3 interface region interact with similar cohorts of E3 domains [9], [13]. Although these studies primarily detected physical E2-E3 interactions, careful comparison between these physical interactions with literature-curated biochemically-functional ubiquitin-conjugating E2-E3 pairs revealed a good degree of overlap [13]. Surprisingly, in our previous study [13], two highly homologous E2 enzymes, UbcH6 (UBE2E1) and UbcH8 (UBE2E2) were identified to show remarkable different E3 interaction patterns. The astonishing difference in the E3 (RING) interaction profiles of those two enzymes, despite their high similarity, is supported by biochemical and functional evidences that suggest a minor role for UbcH8 in ubiquitin-conjugation while UbcH6 is reported as a major ubiquitin-conjugating enzyme [17]. While UbcH6 was shown to interact with approximately 24 RING-finger domains, UbcH8 only contacts two RING E3s [13]. Both E2 enzymes differ within their classical E3 recognition interface in only three residues (D58/E66 in H1 and T103/S111 and E105/D113 in L1) and in a lesser-conserved N-terminal extension of approximately 60 amino acids to the conserved UBC-fold (***Figure 1A***). The observed differences in E3 interaction patterns are therefore in striking contrast with the high levels of sequence identity between these two E2s.

To understand how these minimal variations in amino acids composition can cause divergent E3 interaction patterns, residues in UbcH8 were substituted with their counterparts from UbcH6 and experimentally tested by yeast two-hybrid screening for interaction against 250 human isolated E3-RING domains. In agreement with our previous study [13], wild type (WT) UbcH6 binds 24 E3s while UbcH8 weakly interacts with two RING-finger domains. Interestingly, the UbcH8 E66D and UbcH8 D113E mutations allow interactions in a similar rich profile as UbcH6, even though the sequence alterations were minimal and did not induce any major changes in physicochemical side-chain properties (***Figure 1B*** and ***Supplemental figure S1A***). In contrast, UbcH8 S111T did not display an interaction profile similar to UbcH6, indicating that the amino acid at this position is less crucial in determining E2-E3 selectivity (***Supplemental figure S1A***). Intriguingly, the reverse substitutions, UbcH6 D58E and UbcH6 E105D, did not affect the selectivity profile of UbcH6 (***Supplemental figure S1B***). Thus, minimal differences in side-chain characteristics (the presence of a single methylene moiety) can profoundly change the E3 binding profiles of strictly conserved E2 enzymes.

To investigate the underlying molecular mechanisms at the origin of these differences, the structural environment of the differentiating residues in the unbound structures of both E2 enzymes was evaluated in more detail. Structural analysis of the respective PDB files (UbcH6: PDBid 3BZH; UbcH8; PDBid 1Y6L) revealed that the side-chains of H1 D58/E66 are remote from and not pointing toward the E3 interaction interface. Because of this, we decided to concentrate on the L1 D-to-E substitution. Inspection of UbcH6/UbcH8 E105 and D113 revealed that both amino acids are surface-exposed with their side-chains pointing toward the predicted E2-E3 interaction surface.

To dissect potential effects of the D-to-E substitution at the E2-E3 interface, E2 structures were studied in complex with the E3 RING domain. The RING-finger domain of the ubiquitin ligase TOPORS, previously reported to interact with UbcH6 but not with UbcH8, was used. Since at present, there are no experimental structures of UbcH6 or UbcH8 in complex with a RING domain, we used existing E2-E3 structures as templates, combining three-dimensional alignments, protein threading and refinement in explicit solvent using HADDOCK [18], [19], to generate models of UbcH6-TOPORS and UbcH8-TOPORS that were free of steric clashes or any specific problems within the interface. They both look very similar at the macromolecular level (***Supplemental figure S2***). Interestingly, both E2-E3 structures vary only in their intermolecular electrostatic contributions (UbcH6-TOPORS: −249±28 kcal/mol vs. UbcH8-TOPORS: −195±40 kcal/mol). The model of UbcH6-TOPORS reveals that E105 directly interacts with a positively charged patch on TOPORS, forming an intermolecular salt-bridge with K33 that may stabilize the interaction (***Supplemental figure S2A***). In contrast, the interface between UbcH8 and TOPORS lacks this intermolecular salt-bridge, since the shorter side-chain of D113 in UbcH8 seems unable to interact with K33 in TOPORS (***Supplemental figure S2B***). Further analysis of the intermolecular electroscatic energy component of each amino acid at the interface reveals that the E105/D113 difference is indeed one of the major contributions to the difference in electrostatic energy in the HADDOCK models.

**Figure 2:**
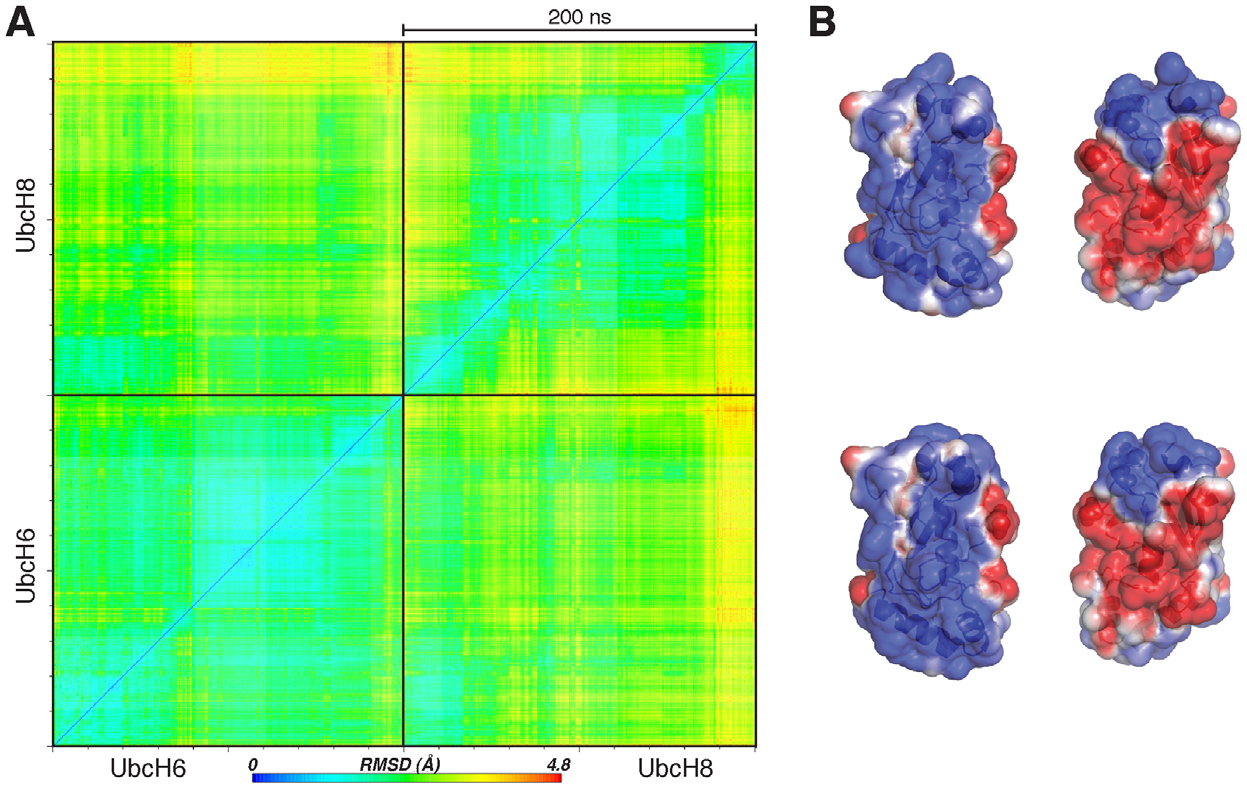
Study of electrostatic potentials and conformational stability of UbcH6 and UbcH8 enzymes. **A**. MD simulation cross profiles of UbcH6 and UbcH8 depicting cross-RMSDs calculated over all E2s Cαs and Cβs in the same trajectory. **B**. Electrostatic potentials mapped onto the solvent accessible surface of UbcH6 and UbcH8. Two views of each protein are shown (low electron density: blue; high electron density: red; neutral: white). The electrostatic potential surfaces were determined using the Adaptive Poisson-Boltzmann Solver package (APBS) [37] within PyMOL [38].

**Figure 3:**
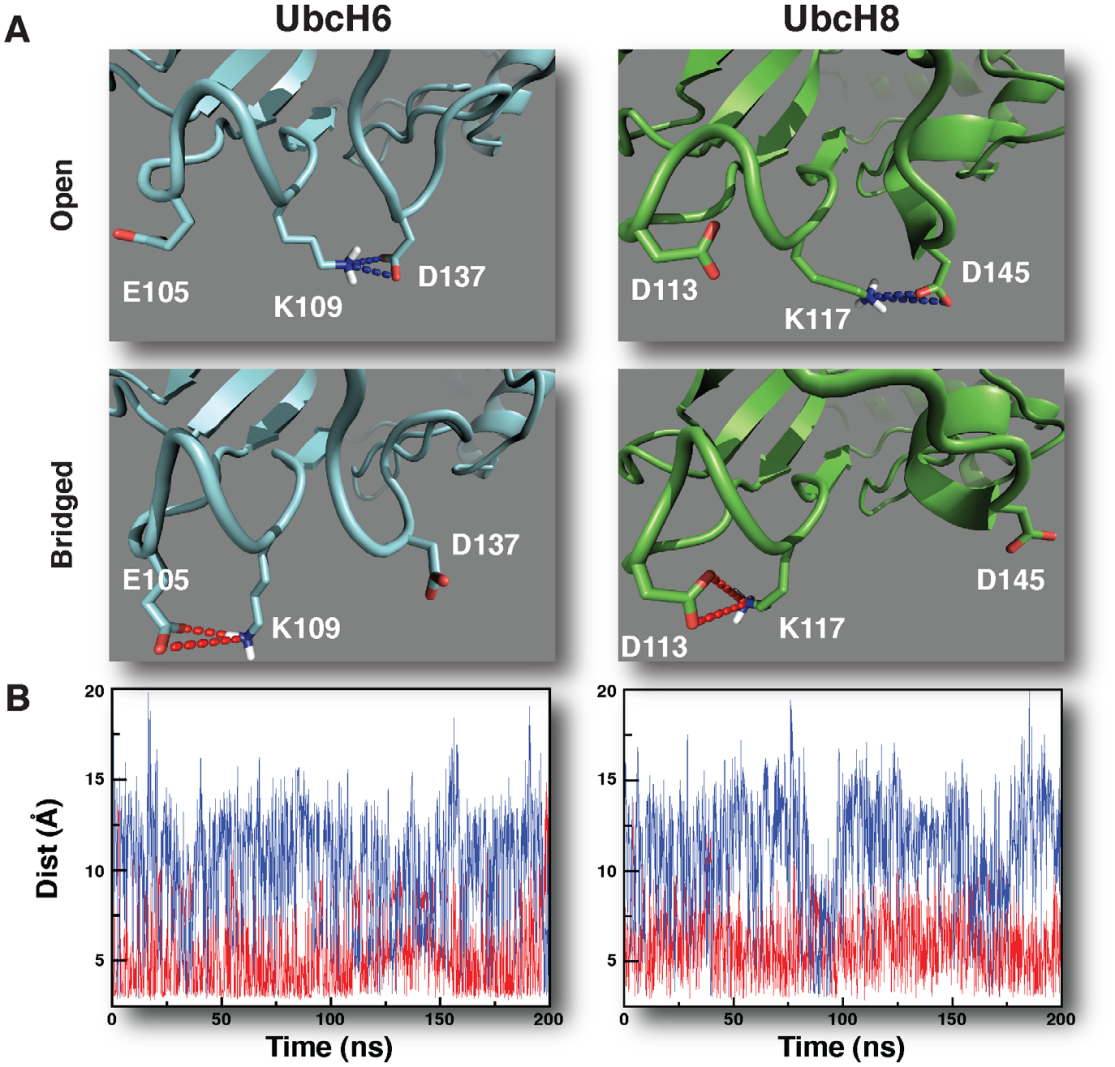
Properties of the E2 intra-molecular salt-bridge network. **A.** Atomic view of the UbcH6/UbcH8 intra-molecular salt-bridge network that controls E105/D113 positioning and E3 binding. Images were generated with the PyMOL Molecular Graphics System, Version 1.3 [38]. **B.** Dynamics of the intra-molecular salt-bridge network. Depicted are E2-specific distances between the indicated side-chains during a 20 ns MD trajectory. Curves were smoothened using a 100 ps running average window.

To further study the impact of the D-to-E substitution, extended molecular dynamics (MD) simulations of the free forms UbcH6 and Ubch8 were performed. Cross-RMSD values indicated no significant conformational changes (***Figure 2A***). Similarly, electrostatic potential maps of UbcH6 and UbcH8 did not reveal any differences, neither for the original structures nor for snapshots extracted from the MD trajectories (***Figure 2B***). These results indicate that the observed changes in E3 interaction patterns upon the D-to-E substitution cannot be explained by simple alterations in electrostatic potentials or major conformational rearrangements of the E2 enzymes. The lack of apparent energetic and/or conformational differences between those E2 enzymes made us look into the internal dynamics within and around their interaction surface. We therefore studied the potential role of neighboring residues on the side-chain conformations of E105/D113. Throughout the molecular dynamics simulations, the distances between surrounding residues and the central E105/D113 were monitored, focusing in particular on a salt-bridge network involving E105/D113 and a number of residues outside the interface. This analysis revealed a subtle equilibrium between two conformations in L1 carrying the E105/D113 residues (***Figure 3A***). First, an *“open”* or *binding-competent* conformation was observed that enables access of the E105/D113 residue for RING-interaction. Second, a *“bridged”* or *binding-impaired state* could be found, in which the side-chain of E105/D113 is hijacked by the adjacent residue K109/K117. As a direct consequence, E105/D113 cannot interact with the RING and points away from the interaction surface. In both UbcH6 and UbcH8, the side-chain of K109/K117, respectively, plays a central role in controlling the equilibrium by forming an intramolecular salt-bridge with E105/D113, respectively. Interestingly, the lysine at this position is highly conserved among other members of the E2 superfamily and has previously been implicated in establishing E2-E3 specificity [10]. K109/K117 plays thus a crucial role in this equilibrium by shuttling between two intra-molecular salt-bridges: one between K109/K117 and E105/D113, resulting in the “bridged” binding-impaired conformation, and the second between K109/K117 and D137/D145, releasing thereby E105/D113 to adopt the “open” binding-competent conformation (***Figure 3A***). Apart from K109/K117, residues D137/D145 are also highly conserved among members of the E2 superfamily. Interestingly, residues D137/D145 and K109/K117 are all remote from the interaction surface, emphasizing that non-interface residues can also be involved in controlling E2-E3 interactions.

The dynamics of this salt-bridge network was further studied for both E2 enzymes by monitoring the distances between E105-K109 and K109-D137 in UbcH6 and D113-K117 and K117-D145 in UbcH8. We observed a slightly higher population of the binding-impaired conformation (D113-K117 salt-bridge formed) in UbcH8 WT than UbcH6 WT. Furthermore, the exchange toward the open form seems to be more frequent in UbcH6 WT (***Figure 3B***). More importantly, this analysis revealed that the distances are anti-correlated: the central K109/K117 is bound either to E105/D113 or to D137/D145 (***Figure 3B***). This was confirmed by calculating the normalized correlation coefficient between the distances E105-K109 (respectively D113-K117) and K109-D137 (respectively K117-D145) as 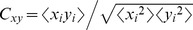. This analysis gives correlation coefficients of −0.82 and −0.89 for UbcH6 and UbcH8, respectively, confirming numerically this anticorrelation of the distances.

The observed differences between the two E2 enzymes are clearly not sufficient to explain the experimental differences in selectivity profile. Still the MD simulations were key in identifying the network components. From these observations, we hypothesized that differences in side-chain length introduced by the D-to-E mutation are able to gently shift the balance of the labile conformational equilibrium between the open (binding-competent) and the bridged (binding-impaired) conformations. Accordingly, the key in controlling E3 interaction profiles between UbcH6 and UbcH8 should reside in the subtle intra-molecular dynamics of residues in L1, which, in turn, are influenced by residues remotely located from the interaction surface that are part of the identified salt-bridge network. If this hypothesis is true, then mutating residues in this network should also have impact on the selectivity profile of these E2s, something that can be experimentally tested.

### Validation of network components

The hypothesis of a side-chain equilibrium controlling UbcH8 and UbcH6 E2 interactions with E3 RING-finger domains was tested experimentally by perturbing key residues involved in the intra-molecular salt-bridge network.

**Figure 4:**
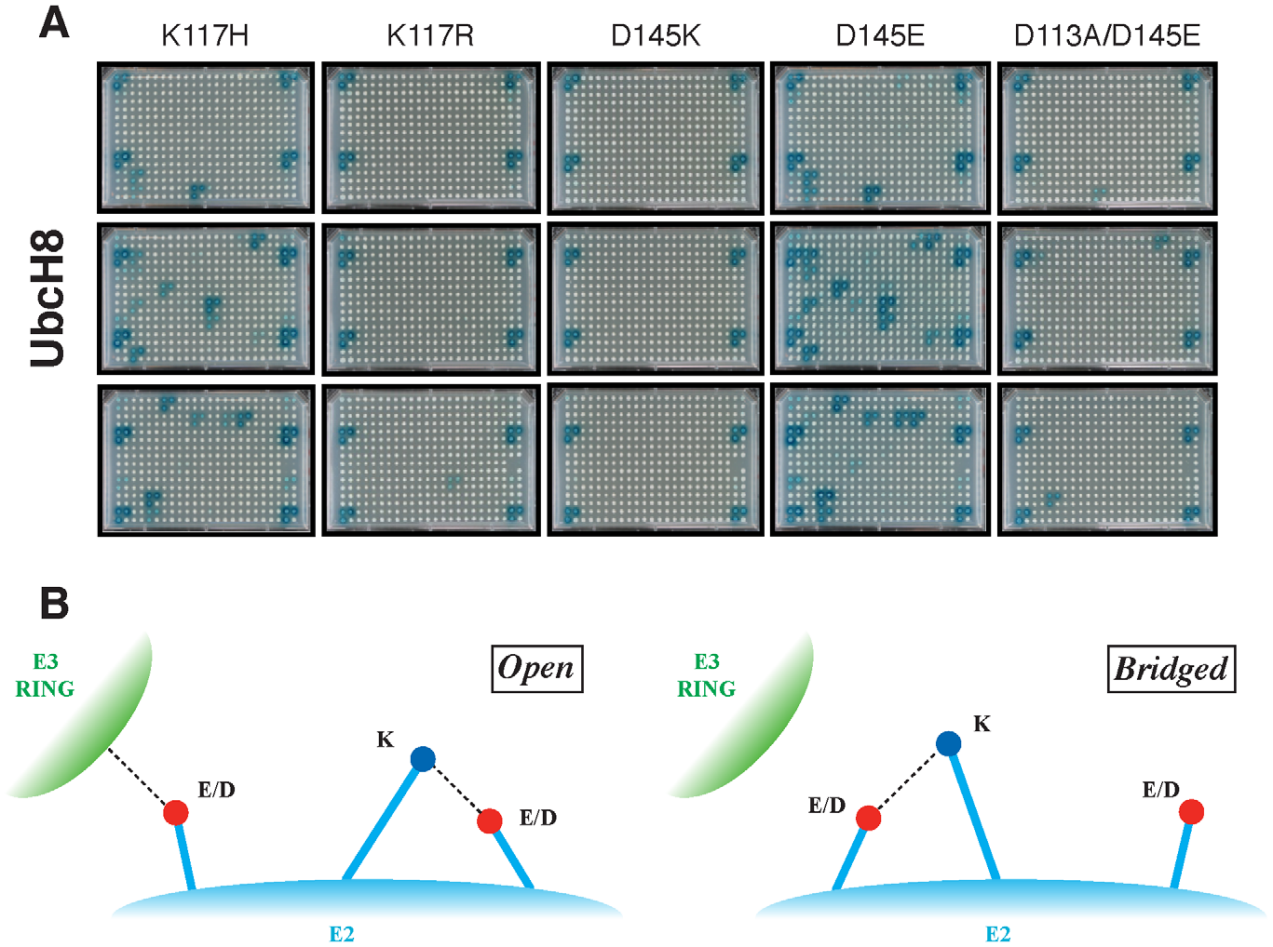
Validation of the dynamic salt-bridge network that controls RING E3 interaction selectivity. **A.** Perturbation of critical network components displaces E105/D113 and controls E3 binding. Mutagenesis of strictly conserved residues that mediate the bridging status of D113 in UbcH8 were mutated to challenge the MD predictions (see main text and Material and Methods section for details). **B.** Schematic representation of the “open” and “bridged” conformations of the intra-molecular network controlling E3 interactions. Indicated are E2-specific residues as well as the conserved network residues that mediate E3 binding.

First, the significance of K117 was assessed in UbcH8, since K117 was predicted to play a central role in alternatively contacting D113 and D145, thereby, preventing D113 of interacting with the RING domain. By substituting UbcH8 K117 with a histidine, the E3 interaction pattern of UbcH8 was reverted to that of UbcH6 (***Figure 4A***). Histidine was chosen in order to maintain the total charge of the system. Note that, since the intracellular pH of yeast has been found to be around 5.5 in most growth phases, it can be expected that the solvent-exposed histidine side-chains are protonated [20]. The result of this substitution is that the positively charged K117 side-chain is replaced by the shorter imidazole side-chain of histidine. This conservative mutation, K117H, does no longer allow a favorable salt-bridge formation with D113 due to the more rigid histidine side-chain, leaving the latter free to interact with the RING domain. This indeed yielded an interaction pattern that resembles that of UbcH6. In contrast, UbcH8 K117R preserves the UbcH8 interaction profile because arginine, which resembles lysine in terms of length and charge, is still able to bridge D113, shielding it away from interacting with the E3 (***Figure 4A***). These experimentally validated observations confirm the central role of K117 of UbcH8 as a regulator of the orientation of D113 and D145 and thereby as a regulator of RING interactions.

We next investigated the role of D145, the most remote (as seen from the E3 interaction site) component of the network. Interaction screening of an UbcH8 D145K mutant demonstrates that D145K interacts in a similar manner as UbcH8 WT (***Figure 4A***). The introduced charge repulsion between D145K and K113 reinforces the bridging between K117 and D113, locking the latter into a “closed” salt-bridged conformation preventing RING-contacts.

In contrast, the introduction of D145E in UbcH8 alters the interaction profile of UbcH8 WT to that of UbcH6 WT. Here, the longer glutamine side-chain can more effectively contact K117, forming a stable salt-bridge, and consequently freeing D113 for forming contacts with RING. To ascertain that this indeed involves D113, the double mutant UbcH8 D113A/D145E was also characterized, showing the loss of the previously gained E3 interactions (***Figure 4A***). These results also indicate that both ASP and GLU at position 113 are binding competent, *provided they are not stabilized in their closed conformation by intra-molecular salt-bridge formation*, highlighting again the key role of this salt-bridge network.

Taken together, these results point toward a delicate network of intra-molecular salt-bridges that dynamically control the positioning of a crucial E3-interacting residue (schematically represented in ***Figure 4B***). Residues located in the classical E2-E3 interface, as well as those that are more distantly located dynamically control the positioning of K117 that acts as a gatekeeper for the negatively charged hot-spot residue capable of mediating E3 interactions. While pH-dependent [21] or phosphorylation [22] regulatory mechanisms have been described previously, the subtle equilibrium identified here does not involve any change in net charge, but originates from a minimal difference of a single methylene group along a side-chain.

## Discussion

Although the catalytic UBC-fold of E2 enzymes is characterized by high levels of sequence and structural similarities, it is adapted to selectively recognize RING-finger domains to allow transfer of Ub to substrates [2], [23]. By comparing sequence and structure of two highly similar E2 enzymes with their global RING-finger interaction patterns, in combination with molecular dynamics simulations, we were able to identify an intra-molecular network of salt-bridges that actively control RING interactions. Perturbation of components of this dynamic network in combination with Y2H screening of interactions subsequently confirmed its crucial role in controlling the interaction specificity. Residue K109/K117 was identified to play a crucial role within this network. It binds in an exclusive manner either E105/D113 or D137/D145. This lysine appears to be strictly conserved among E2 enzymes. In UbcH5B, for example, the corresponding lysine residue at this position is the central K63, which has been intimately linked to UbcH5B selectivity within the UbcH5B-CNOT4 RING-interaction [15], [24]. Interestingly, UbcH5B K63E failed to interact with the WT CNOT4 RING-finger domain, but this could be fully restored by incorporating the charge-swapping D48K/E49K substitutions in CNOT4 [15]. Although K63 interacts directly with D48/E49, this is not the case for K109/K117, indicating that even among conserved residues there are different ways of involvement in generating selectivity and E2-E3 pairing. Additionally, the most remote component of the identified salt-bridge network, D137/D145 is also conserved in the E2 superfamily, but it is not directly involved in RING interactions. The demonstration in this work that mutations at this position can switch selectivity emphasizes that E2-E3 interaction specificity can be a consequence of a subtle, dynamic interplay between interface and non-interface residues.

In the majority of E2 enzymes, residues involved in establishing E3 interaction specificity are not concentrated in a single hotspot, but dispersed over the N-terminal helix one and two relatively flexible and divergent loop regions (L1 and L2) [2], [11]. Mutagenesis of these residues can abolish or modulate E2-E3 specificity [10], [11], [15]. Apart from sequence information, additional structural characteristics are affecting RING selectivity, like the length of H1, the flexibility of L1 and L2 and the triangular distances between these elements [12], [24], [25]. Comparing the sequences of UbcH6 and UbcH8 reveals additional amino acid differences at several key positions in these regions. One of them, D58/E66, lies within H1 and it is not orientated toward the E2-E3 interface. D58/E66 is in close proximity to T144/152 and S146/154, which are solvent exposed and directly interacting with RING domains. Therefore, D58/E66 might affect E3 interactions either indirectly through T144/152 and S146/154 or via a repositioning of helix H1. Indeed, the linker region that connects H1 with the rest of the E2 enzyme is found to be flexible [26] and D58/E66 might be involved in a hinge-like mechanism that might allow some conformational freedom of H1, which influences the distances between the three triangle points and thereby RING-interactions.

Finally, the N-terminal extension of the tested E2 enzymes, not present in the crystal structures, might also affect their E3 binding preference. This could explain why reverse mutations in UbcH6 that mimic UbcH8 did not restore the E3 interaction profile of UbcH8. The presence of another intermolecular salt-bridge in the UbcH6-TOPORS complex generated by HADDOCK, involving the residues LYS43 (UbcH6) and GLU28 (TOPORS) (data not shown), could explain why UbcH6 E105D did not affect the selectivity profile of UbcH6. This salt-bridge cannot be formed with UbcH8, where LYS43 is replaced by ALA51 in the sequence. Comparing the sequences of UbcH6 and UbcH8 indeed reveals amino acid differences at several key positions in this N-terminal extension known to be primarily involved in the interaction with the E1 ubiquitin-activating enzyme, but also to play a role in E3 interaction selectivity.

We should note that Y2H screening of physical protein-protein interactions between E2 enzymes and E3 RING-finger domains does not directly address biological functionality, e.g. the ability to transfer the activated Ub. However, we previously demonstrated that E2-RING E3 interactions found by LexA-B42 yeast two-hybrid assays are good predictors for enzymatic functionality [13] and therefore believe that the results presented here are also relevant in a functional context. It is unlikely that minor alterations in side-chain characteristics described in this work would affect protein stability *in vivo* (and our *in silico* results are supporting this). In addition, a recent systematic analysis of interaction dynamics across different technologies reported that high-throughput yeast two-hybrid is the only available technology for detecting transient interactions on a large scale [27], which support the recourse to this technique to unravel labile E2-E3 interactions.

Despite their high percentage of identity, it has been reported that UbcH6 plays a major role in ubiquitin-conjugation while UbcH8 has only a minor role in ubiquitination but rather is the key conjugating enzyme of the ISGylation pathway [17]. This functional difference is in line with our observations [13] and supports the importance of an E3 selectivity mechanism that must differenciate not only among Ub-E3 ligases but also between Ub and UbL E3 ligases. Finally, this unreported dynamic intra-molecular salt-bridges network constitutes a new fundamental principle to understand the structural and evolutionary determinants of multispecific recognition.

In conclusion, a dynamic equilibrium of conserved residues in two highly homologous E2 enzymes was identified, that mediate RING interactions. Amino acids located both within the classical interaction surface as well as residues that are remote from this surface are actively involved in modulating side-chain conformations and thus availability for binding of crucial residues. The subtle and dynamic nature of the identified regulatory switch suggests new ways how protein interactions can be controlled. Furthermore, the observation that minimal sequence differences between two highly similar proteins can control protein interaction networks serves as a cautionary tale and raises new challenges for bioinformatics analysis and modeling of protein interactions. Finally, these findings indicate that the widely accepted sequence-structure-function paradigm should be extended to *sequence-structure-dynamics-function* relationship.

## Materials and Methods

### Plasmids and mutagenesis

High-copy yeast-two hybrid (Y2H) shuttle plasmids expressing human UbcH6 (UBE2E1) and UbcH8(UBE2E2) and RING-finger domains of 250 human RING-type E3 ubiquitin protein ligases as fusions with the *E. coli* LexA binding domain (BD) or with the B42 acidic activator domain (AD), respectively are described in [13]. All amino acid substitutions were introduced using QuickChange II Site-Directed mutagenesis (Stratagene), according to the manufacturers' instructions. Appropriate mutagenesis was validated using DNA sequencing.

### Molecular modeling

Both wild-type and mutant UbcH6 and UbcH8 were used as starting structures. Wild-type UbcH6 and UbcH8 structures were taken from the Protein Data Bank (PDB) (PDB-ID *3bzh* and *1y6l*, respectively). Selective mutations were introduced with CNS (Crystallographic and NMR System) [28], keeping the Ca and the Cb atoms fixed to preserve the side-chain rotamer. Each system was simulated in duplicate for 200 ns using the GROMOS G53a6 force field [29] and GROMACS 4.5.5 package [30]. The starting structures were solvated in a dodecahedral box with a minimal 14 Å distance between solute and box, resulting in systems of about 11500 SPC water molecules, 34 Na^+^ and 36 Cl^−^ ions (±1, depending on the system). Simulations were performed under periodic boundary conditions at 300 K and constant pressure (1 bar), using either Berendsen [31] or v-rescale [32] coupling algorithms for temperature control and Berendsen [31] coupling algorithm for pressure control (with coupling constants of 0.1 and 1.0 ps, respectively). Bond lengths were constrained with the Linear Constraint Solver (LINCS) algorithm [33] and the time step for the integration was 2 fs. Electrostatic interactions were calculated using either the reaction-field method [34] with a 14 Å cut-off distance or Particle Mesh Ewald (PME) [35], [36] with a 10 Å cut-off distance. Non-bonded interactions were updated every 10 fs (with a 10 Å cut-off distance for the short-range neighbor list). MD analysis was performed excluding the first 1 ns of the trajectories. All various simulation settings resulted in similar trajectories and only the results obtained with PME and v-rescale temperature control are discussed here.

### Structural alignment and refinement of E2-E3 complexes with HADDOCK

Structural models of UbcH6 and UbcH8 in complex with the RING-finger domain of TOPORS were generated by structural alignment of single components onto the bound structures of existing E2-E3 complexes (PDB: *1fbv*, *1ur6*, *3eb6*, *2c2v* and *2oxq*). The resulting models were subjected to a short refinement in explicit water using HADDOCK [18], [19].

### Electrostatic potentials of E2 enzymes

Electrostatic potential surfaces were determined using the Adaptive Poisson-Boltzmann Solver package (APBS) [37] within Pymol (version 1.3) [38].

### Yeast two-hybrid analysis

Manipulation of yeast cells and two-hybrid techniques are described in [13]. Briefly, WT and mutant LexA-E2 fusions were co-transformed together with the pSH18-34 LacZ reporter plasmid in EGY48α cells. To evaluate the effects of E2 mutants on human RING-type interactions, 250 sequence-verified B42-RING constructs were arrayed in EGY48a cells. Mating between α and A cells was performed on non-selective Yeast Peptone Dextrose (YPD)-medium (24 hours at 30°C). Diploids were selected for 48 hours on synthetic complete (SC) medium lacking the amino acids histidine, tryptophan and uracil (HWU^−^ medium) by manual transfer of yeast spots using a 384-well replicator pinning tool (V & P Scientific, San Diego). Putative interactions were assessed in triplicate by growing diploids on SC HWU^−^ medium supplemented with 5-bromo-4-chloro-3-indolyl β-D-galactopyranoside (X-gal) or on SC HWUL^−^ (idem as HWU^−^ but also lacking leucine), including either galactose or glucose as main carbon source. Quantification of interactions was done after 72 hours as reported earlier [13].

## Supporting Information

Figure S1 **Effects of reverse substitutions for UbcH8 and UbcH6 determined on global patterns of E3 interactions.** **A.** E3-interaction profiles of UbcH8 E66D and S111T. **B.** E3-interaction profiles of UbcH6 D58E and E105D.
(TIF)

Figure S2 **Models of UbcH6 and UbcH8 bound to the E3 RING-finger TOPORS domain.** **A.** Cartoon representation of the UbcH6-TOPORS model (UbcH6: cyan; TOPORS: magenta). **B.** Cartoon representation of the UbcH8-TOPORS model (UbcH8: green; TOPORS: magenta). Images were generated using the PyMOL Molecular Graphics System, Version 1.3 [38].
(TIF)
