# Supplementary figures and images for: Dynamic Control of Selectivity in the Ubiquitination Pathway Revealed by an ASP to GLU Substitution in an Intra-Molecular Salt-Bridge Network
Source: PLoS Comput Biol. 2012 Nov 1;8(11):e1002754. doi: 10.1371/journal.pcbi.1002754 (PMC3486841; doi:10.1371/journal.pcbi.1002754)

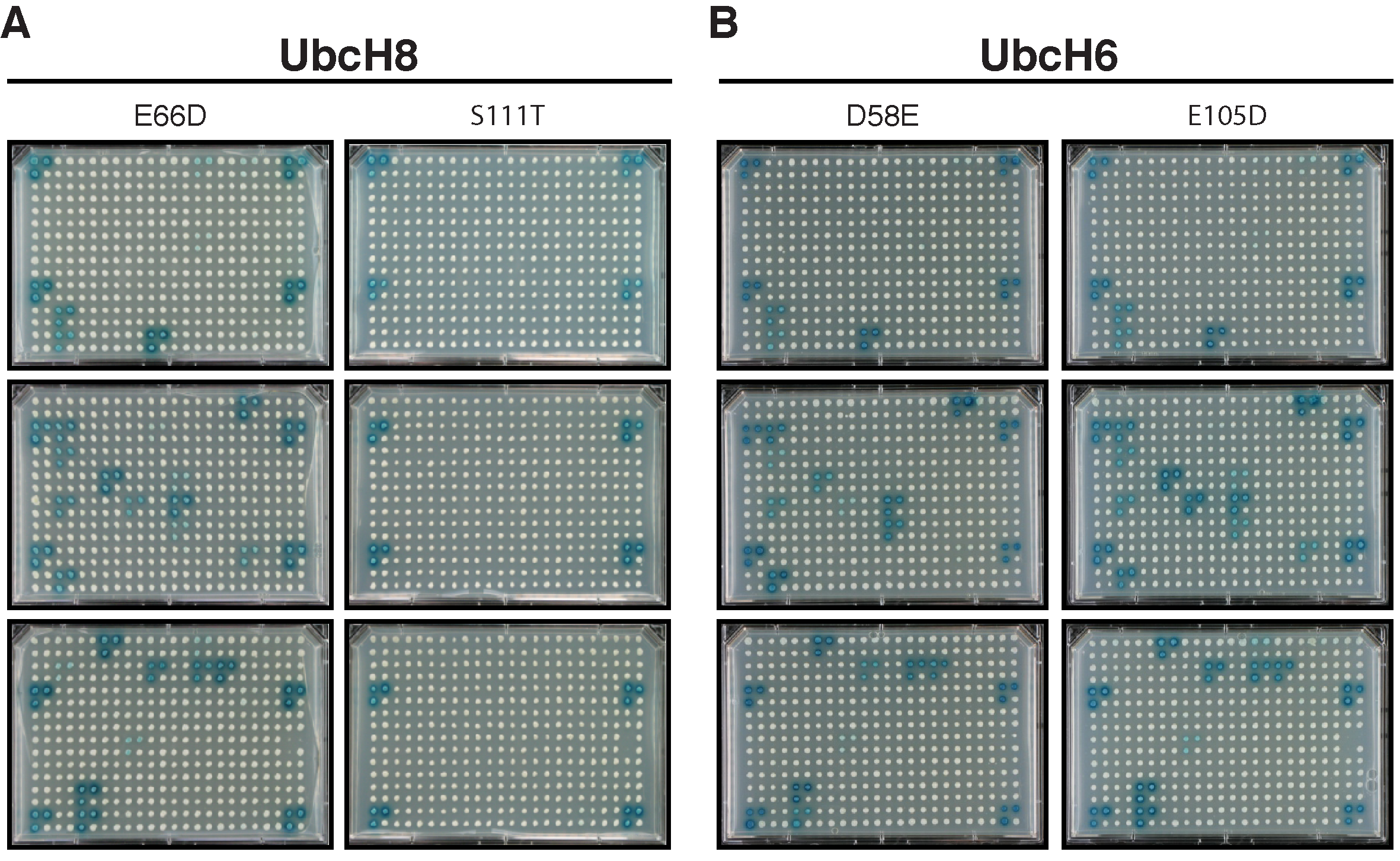

Supplement: Figure S1 — Effects of reverse substitutions for UbcH8 and UbcH6 determined on global patterns of E3 interactions. A. E3-interaction profiles of UbcH8 E66D and S111T. B. E3-interaction profiles of UbcH6 D58E and E105D. (TIF) [file pcbi.1002754.s001.tif]

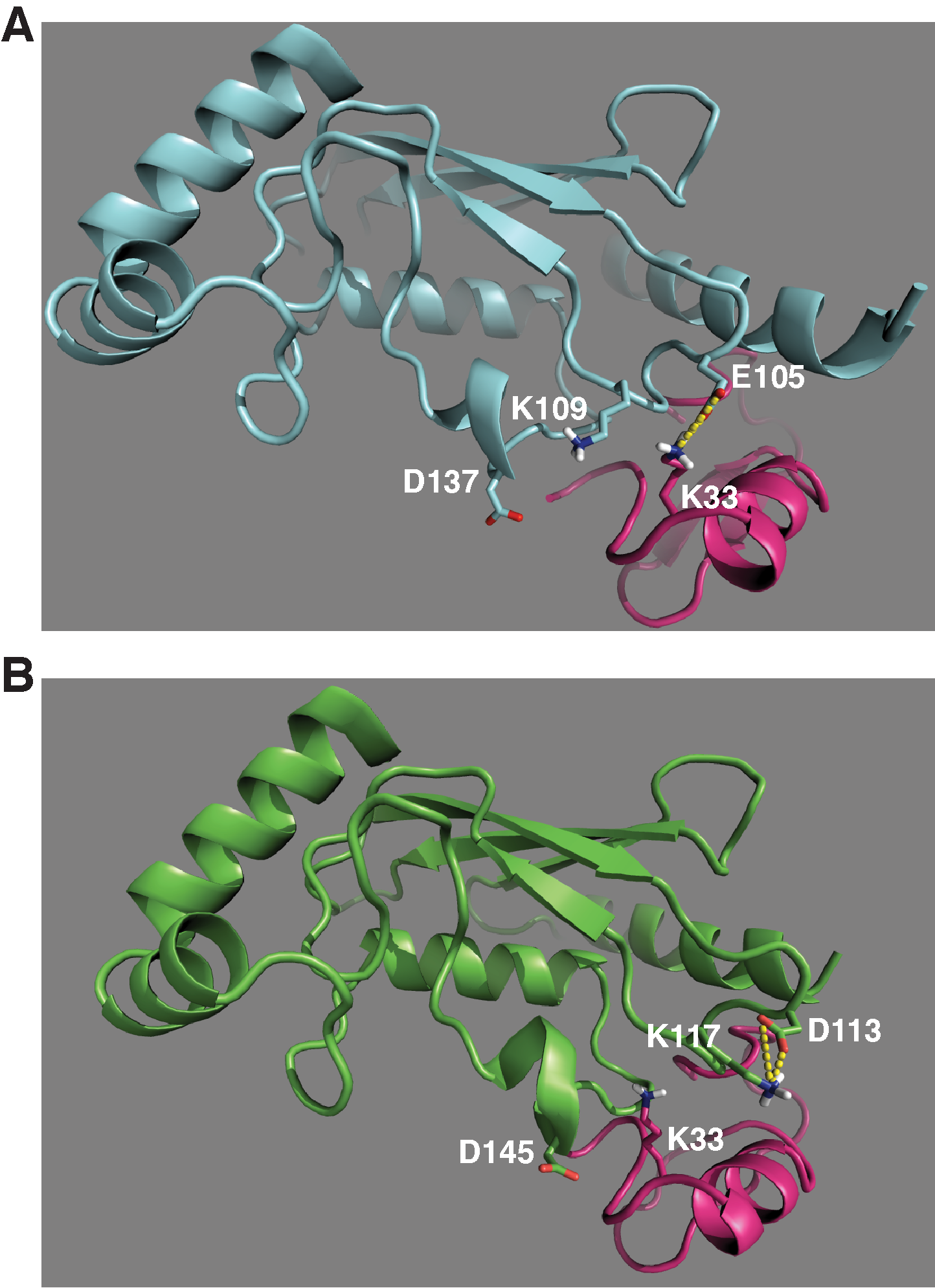

Supplement: Figure S2 — Models of UbcH6 and UbcH8 bound to the E3 RING-finger TOPORS domain. A. Cartoon representation of the UbcH6-TOPORS model (UbcH6: cyan; TOPORS: magenta). B. Cartoon representation of the UbcH8-TOPORS model (UbcH8: green; TOPORS: magenta). Images were generated using the PyMOL Molecular Graphics System, Version 1.3 [38]. (TIF) [file pcbi.1002754.s002.tif]
